# Systemic inflammatory status predict the outcome of k-RAS WT metastatic colorectal cancer patients receiving the thymidylate synthase poly-epitope-peptide anticancer vaccine

**DOI:** 10.18632/oncotarget.24993

**Published:** 2018-04-17

**Authors:** Pierpaolo Correale, Cirino Botta, Nicoletta Staropoli, Valerio Nardone, Pierpaolo Pastina, Cristina Ulivieri, Claudia Gandolfo, Tatiana Cosima Baldari, Stefano Lazzi, Domenico Ciliberto, Rocco Giannicola, Antonella Fioravanti, Antonio Giordano, Silvia Zappavigna, Michele Caraglia, Pierfrancesco Tassone, Luigi Pirtoli, Maria Grazia Cusi, Pierosandro Tagliaferri

**Affiliations:** ^1^ Unit of Medical Oncology, Grand Metropolitan Hospital Bianchi Melacrino Morelli, Reggio-Calabria, Italy; ^2^ Medical Oncology Unit, AUO Mater Domini, Magna Graecia University, Catanzaro, Italy; ^3^ Department of Experimental and Clinical Medicine, Magna Graecia University , Catanzaro, Italy; ^4^ Unit of Radiotherapy, Department of Surgery, Medicine and Neurological Science, Siena University Hospital, Siena, Italy; ^5^ Department of Science of Life, Siena University, Siena, Italy; ^6^ Microbiology and Virology Unit, Department of Medical Biotechnology, Siena University, Siena, Italy; ^7^ Unit of Pathology, Department of Surgery, Medicine and Neurological Science, Siena University Hospital, Siena, Italy; ^8^ Unit of Rheumatology, Department of Clinical Medicine and Immunologic Sciences, University of Siena, Siena, Italy; ^9^ Department of Biotechnology, Temple University, Sbarro Foundation, Philadelphia, Pennsylvania, USA; ^10^ Department of Precision Medicine, University of Campania L. Vanvitelli, Naples, Italy

**Keywords:** bio-markers, cancer vaccine, colorectal cancer, K-ras, thymidylate synthase

## Abstract

TSPP is an anticancer poly-epitope peptide vaccine to thymidylate synthase, recently investigated in the multi-arm phase Ib TSPP/VAC1 trial. TSPP vaccination induced immune-biological effects and showed antitumor activity in metastatic colorectal cancer (mCRC) patients and other malignancies. Progression-free and overall survival of 41 mCRC patients enrolled in the study correlated with baseline levels of CEA, immune-inflammatory markers (neutrophil/lymphocyte ratio, CRP, ESR, LDH, ENA), IL-4 and with post-treatment change in p-ANCA and CD56^dim^CD16^bright^NKs (*p* < 0.04). A subset of 19 patients with activating k-ras mutations showed a different immune-inflammatory response to TSPP as compared to patients with k-ras/wt and a worse outcome in term of PFS (*p* = 0.048). In patients with k-ras/mut, inflammatory markers lost their predictive value and their survival directly correlated with the baseline levels of IL17/A over the median value (*p* = 0.01). These results provide strong hints for the design of further clinical trials aimed to test TSPP vaccination in mCRC patients.

## INTRODUCTION

Thymidylate-synthase poly-epitope peptide (TSPP) is a 27-mer vaccine construct which contains the amino-acidic sequences of three known cytotoxic-T-lymphocyte (CTL) epitopes with HLA-A(*)02.01 amino-acid anchorage motifs (TS-1, TS-2, and TS-3) derived from the thymidylate synthase (TS) and showing promising antitumor activity in preclinical models [1–4]. TS is a cancer-associated target enzyme, critical for DNA replication and repair, commonly overexpressed in proliferating cancer cells, and inhibited by 5-FU metabolites and other anti-cancer drugs [1–3].

Several studies suggest that cytotoxic drugs like oxaliplatin, gemcitabine, 5-FU or cyclophosphamide, mAbs to EGFR and VEGF, as well as radiotherapy, may induce immunogenic cell death, and shape mCRC micro-environmental conditions making the residual tumor tissue more sensitive to activated immune-effectors. On these bases, our group investigated the antitumor activity and the toxicity of the TSPP vaccination alone and in combination with 5-FU in HLA-A(*)02.01 (HHD) transgenic mice inoculated subcutaneously with TS-expressing EL-4/HHD lymphoma cells. In these *in vivo* models, TSPP was able to elicit a specific CTL response without inducing autoimmunity or toxicity and showed a higher antitumor activity in combination with 5-FU [2]. Successively, TSPP was tested in *a* multi-arm phase Ib (TSPP/VAC-1) trial in metastatic pretreated cancer patients with different malignancies, including metastatic colorectal carcinoma (mCRC) and non-small-cell-lung-cancer (NSCLC) [5, 6]. The trial investigated the effects of TSPP vaccination alone (arm-A) or associated with granulocyte macrophage-colony-stimulating-factor (GM-CSF) and interleukin-2 (IL-2) (arm-B) [5, 7], or in concomitant (Arm-C/DL1-3) or sequential (arm-C/DL0) combination with a previously characterized [5, 6] chemo-immunotherapy regimen with gemcitabine, oxaliplatin, levo-folinic acid (FA) and bolus/infusional 5-FU followed by IL-2 and GM-CSF (GOLFIG regimen) [6, 8–11]. The study enrolling 50 patients (12 in the arm A, 9 in the arm B, and 29 in the arm C) showed that TSPP is safe (MTD was not achieved), exerts immune-modulating effects and produces self-limiting auto-immunity signs in all of the experimental arms. Specifically, TSPP vaccination elicited TS-specific-T cell response and was associated to a progressive rise in serum auto-antibodies [Anti-nuclear anti-bodies (ANA), anti-extractable nuclear antigen (ENA), anti-neutrophil cytoplasmic antibodies/anti-proteinase-3 (pANCA), and anti-myeloperoxidase (cANCA)] [5, 6]. This study reported preliminary evidence of TSPP antitumor activity on mCRC patients representing the majority of patient population [5, 6] and granting the rationale to design further phase II trials. We, therefore, attempted to identify potential biomarkers predictive of treatment response to TSPP by carrying -out a retrospective analysis on a cohort of 41 mCRC patients enrolled in the TSPP/VAC1 trial.

## RESULTS

### Demographics, chemo-immunotherapy, peptide vaccination and dose-escalation

Forty-one mCRC patients, enrolled in the TSPP/VAC1 multi-arm phase-Ib trial between May 2011 and July 2013, were considered for this study. TSPP resulted safe and able to induce immune-modulatory activity, including changes in serum levels of multiple inflammatory markers (CRP, ESR, LDH/NV), Auto-antibodies (ANA, ENA, p-ANCA, and c-ANCA) and TSPP-specific CTL precursors’ frequency. Th1/Th2/Th17 cytokine profile, blood cell counts, peripheral lymphocyte subsets, DCs and MDSCs showed minimal difference among the three study arms. The population of mCRC patients enrolled in the trial was homogeneous in term of clinical and immune-biological features and previous treatments (Table 1). All patients were required to present high tumor expression of TS at baseline, while a subgroup of19 patients (46.3%) also presented an activating k-ras mutation.

**Table 1:** Demographic, clinical and molecular pathology characteristics of the patients enrolled in the clinical trial

CODE	Sex	ECOG	Metastatic sites	Previous treatment lines	K-ras	HLA	Treatment courses	MANT	Type of response
MFA/A/DL1	F	0	Liver, lung	11	Wt	**A2**	3	Na	SD
RAA/A/DL1	F	0	Peritoneum, nodes	7	Wt	**A2**	3	Na	PD
ASA/A/DL1	M	0	Liver, Lung	3	Wt	**A2**	3	Na	SD
LVA/A/DL2	M	0	Liver, Lung, nodes	7	Wt	A11	6	Na	SD
SGA/A/DL2	M	0	Peritoneum, nodes	4	Wt	A25	6	Na	SD
ZSA/A/DL3	F	0	Nodes, Adrenal	3	Wt	**A2**	48	Na	PR
PNA/A/DL3	F	0	Lung, bones	3	Mut	A1	3	Na	SD
SNA/A/DL3	M	2	Liver, lung	3	Mut	**A2**	3	Na	PD
GL/B/DL2	F	0	Liver, abdomen	3	Mut	A24	3	Na	PD
PP/B/DL2	F	0	Lung, liver abdomen	3	Wt	**A2**	3	Na	SD
FG/B/DL2	M	0	Abdomen, nodes	3	Wt	A24	3	Na	PD
MM/B/DL3	M	1	Lung, liver abdomen	3	Mut	A1	3	Na	SD
LM/C/DL1	F	2	Peritoneum, lung, liver	2	Mut	A24	7	2	SD
CV/C/DL1	M	1	Peritoneum, lung, liver	6	Mut	A3	6	0	PD
BF/C/DL1	M	2	Peritoneum, lung, liver	3	Mut	A11	3	0	PD
CL/C/DL2	F	0	Peritoneum, nodes	3	Mut	**A2**	12	1	PD
FA/C/DL2	F	2	Colon, lung, liver	6	Wt	A11	11	0	SD
SA/C/DL2	M	0	Lung, liver, nodes	5	Wt	**A2**	12	2	PR
SS/C/DL3	F	0	Brain, lung, liver, nodes	5	Mut	A23	12	3	PR
BA/C/DL3	F	1	Colon, lung, liver, nodes	2	Mut	**A2**	11	0	SD
SA/C/DL3	F	2	Peritoneum, lung, liver	5	Mut	**A2**	12	0	PD
DA/C/DL3	F	2	Peritoneum, lung, liver	4	Mut	A1	10	0	PD
MP/C/DL3	M	1	Peritoneum, lung, liver	6	Mut	**A2**	6	0	SD
PM/C/DL3	M	1	Peritoneum, lung, liver	2	Mut	A11	12	3	SD
MU/C/DL3	F	1	Soft tissue, nodes	2	Wt	A24	12	14	SD
PA/C/DL3	M	1	Peritoneum, lung, liver	7	Wt	**A2**	11	3	PR
CA/C/DL3	M	2	Peritoneum, lung, liver	3	Mut	NA	6	0	PD
BG/C/DL3	F	0	Peritoneum, lung, liver	3	Wt	**A2**	10	8	SD
NR/C/DL3	F	2	Peritoneum, lung, liver	2	Mut	**A2**	3	0	PD
SR/C/DL0	M	0	Lung, liver	3	Wt	A23	12	5	SD
DG/C/DL0	F	0	Lung, liver	3	Wt	A33	10	5	PR
BL/C/DL0	F	0	Lung, liver	2	Wt	A3	10	9	SD
ML/C/DL0	F	0	Peritoneum, lung, liver	2	Mut	**A2**	10	9	SD
VP/C/DL0	M	1	lung, liver	2	Wt	**A2**	7	1	PD
PG/C/DL0	M	1	lung, liver	2	Wt	A24	9	12	SD
SA/C/DL0	M	2	Peritoneum, lung, liver	3	Wt	A1	7	0	PD
MA/C/DL0	M	0	lung, liver	2	Mut	A1	9	32	PR
PA/C/DL0	M	0	lung, liver	2	Wt	A3	11	14	PR
DL/C/DL0	F	0	Peritoneum, lung, liver	3	Wt	**A2**	9	4	SD
VC/C/DL0	M	0	Peritoneum, lung, liver	2	mut	A23	10	7	PR
PA/C/DL0	F	1	Peritoneum, lung, liver	3	Wt	A23	9	4	SD

Legend: ECOG, Eastern Cooperative Oncology Group performance status; Wt, Wild type; Mut, Mutated; SD, Stabilized Disease; PR, Partial Response; PD, Progressive Disease; MANT, Maintainance Therapy duration in months; Na; not achieved.

### Pathology study

**Figure 1:**
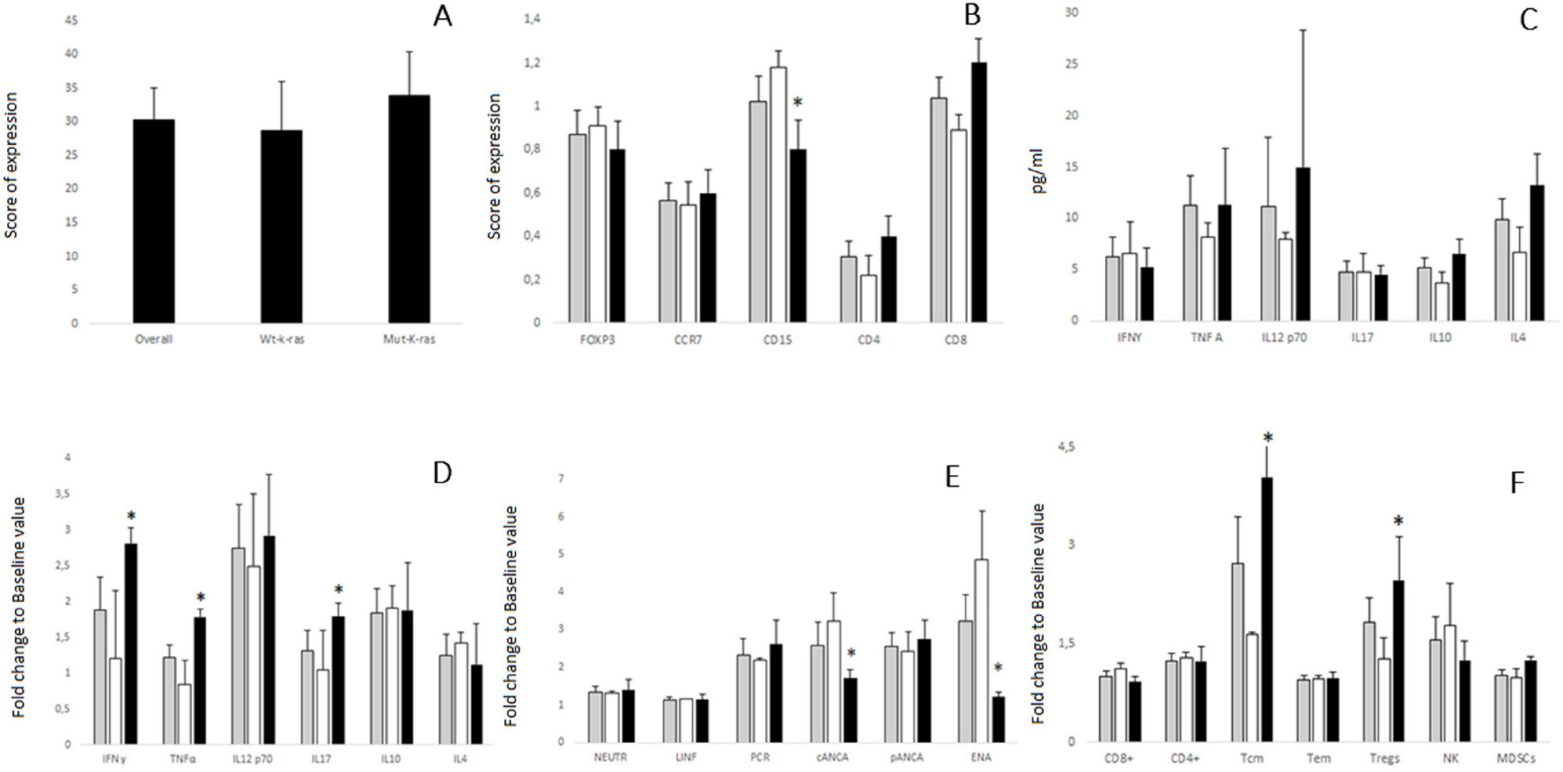
(**A**) Immuno-histochemical analysis of TS expression in the primary tumor of 41 mCRC patients enrolled in the TSPP/VAC-1trials [All patients (overall), patients with k-ras wt (wt-k-ras) and patients with mutated k-ras (mut-k-ras)]. Results are expressed as number of positive cells per HPF ( ± SE). No difference in TS expression was detected between the two subsets of patients. (**B**) Immuno-histochemical analysis of tumor infiltrating T cells expressing FoxP3 (T_reg_), CCR7 (T_cm/em_), CD4, or CD8 and inflammatory cells expressing CD15. This analysis was carried out in the primary tumor of 41 mCRC patients who received TSPP vaccine [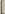], whose 22 with k-ras wt [□] and 19 with mutated k-ras [■]. Results are expressed as number of positive cells per HPF ( ± SE). No differences were detected between the two subsets of patients with exception of CD15^+^ cells, which showed a reduced expression in patients with k-ras mut (*P* = 0.046), Asterisk (^*^) represents statistically significant difference. (**C**) Cytokine Multiplex analysis- Evaluation of baseline serum levels of IFNɣ, TNFα, IL12p70, IL17/A, IL10, IL4 of 41 mCRC patients who received TSPP vaccine 41 mCRC patients who received TSPP vaccine [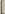], wt [□] and 19 with mutated k-ras [■]. Results are pg/ml ( ± SE). No differences were detected between the two subsets of patients at baseline. (**D**) Evaluation of fold change to baseline values of serum levels of IFNɣ, TNFα, IL12p70, IL17/A, IL10, and IL4 of 41 mCRC patients who received TSPP vaccine [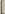], whose 22 with k-ras wt [□] and 19 with mutated k-ras [■]. Results are expressed as fold induction relative to baseline indicated as 1 ( ± SE). Asterisk (^*^) represents statistical significance to between k-ras mut vs k-ras wtpatients (*P <* 0.05); hashtag (^#^) represents statistical significance to baseline value (*P <* 0.05). (**E**) Evaluation of fold change to baseline values of Neutrophils, lymphocytes, CRP, cANCApANCA and ENA of 41 mCRC patients who received TSPP vaccine [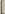], whose 22 with k-ras wt [□] and 19 with mutated k-ras [■]. Results are expressed as fold induction relative to baseline indicated as 1 ( ± SE). Asterisk (^*^) represents statistical significance to between k-ras mut vs k-ras wt patients (*P <* 0.05); hashtag (^#^) represents statistical significance to baseline value (*P <* 0.05). (**F**) Flow cytometry- Evaluation of fold change to baseline levels of peripheral blood cells expressing the following phenotypes: CD3^+^CD4^+^, CD3^+^CD8^+^, or CD8^+^CD45Ra^-^CCR7^+^ (T_cm_s), CD8^+^CD45Ra^-^CCR7^-^ (T_em_s), CD3^+^CD4^+^FoxP3^+^ (T_reg_s), CD3^+^CD56^dim^CD16^bright^(cytotoxic NK), and myeloid derivative suppressive cells (MDSCs). This analysis was performed on 41 mCRC patients who received TSPP vaccine [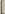], whose 22 with k-ras wt [□] and 19 with mutated k-ras [■]. Results are expressed as fold induction relative to baseline indicated as 1 ( ± SE). Asterisk (^*^) represents statistical significance to between k-ras mut vs k-ras wt patients (*P <* 0.05); hashtag (^#^) represents statistical significance to baseline value (*P <* 0.05).

An immune-histochemical primary tumor analysis in patients bearing wild type k-ras (k-ras/wt) (22 cases) or activating K-ras mutations (K-ras/mut) (19 cases) prior vaccination, revealed no significant difference concerning TS expression (score of expression: 30 for overall, 28 for K-ras/wt and 33 for K-ras/mut ) (Figure 1A) and tumor infiltration by CD4^+^, CD8^+^, CCR7^+^ T cells and T_reg_s (Figure 1B). A lower infiltration score of inflammatory CD15^+^cells was conversely found in patients with k-ras/mut (Figure 1B). It was also observed a significant post-treatment decrement in TS expression in the tumor samples of 10 patients undergone biopsy or surgery after multiple TSPP vaccinations [baseline *versus* (vs.) post-treatment values: 30.2 ± 4.69 *vs.* 3.7 ± 0.98; *P* = 0.011). Other post-treatment correlations (tumor infiltration by immune-cell subsets and k-ras stratification) could not be performed for inadequacy of biological material.

### Immune-monitoring

Our immune-biological analysis did not reveal differences between patients with k-ras/wt and k-ras/mut, in term of inflammatory markers, peripheral immune-cell subsets and cytokine immune-profile at baseline (Figure 1C–1F). There was a general rise in CRP, c-ANCA, p-ANCA, ENA, and T_reg_S and T_cm_s, IFNɣ, IL12/A, and IL10 in the whole patients’ population (Figure 1D–1F). On the other hand, patients with k-ras/mut showed greater and progressive rise in the peripheral levels of IFNγ, TNFα, and IL17/A, a significant increase in peripheral T_cm_s, and T_reg_s, and no change in the levels of cANCA and ENA, which conversely, resulted largely increased in patients with k-ras/wt (Figure 1D–1F). No significant differences were recorded for NEUTR, LINF, CD4+, CD8+,and T_em_s.

### Patients’ outcome correlations

The impact of these inflammatory and immunological parameters (Table 2) on the outcome of TSPP vaccinated patients was also investigated. Altogether, these patients presented an OS of 14.902 ± 2.575 (95% CI 9.85–19.85) months, with 13 out of 41 cases surviving more than 12 months. In our series, PFS and OS were inversely correlated with the baseline performance status (ECOG score) and CEA levels. Patients with k-ras/mut presented a worse outcome with shorter PFS (Table 3) and no differences in OS. We did not find significant differences when PFS and OS were correlated with the number of previous treatment lines, age, gender, HLA-A(*)02.01 haplotype, and TS-and immune-cell tumor infiltration scores, TS score change after treatment (data not shown).

**Table 2:** Immunophenotypic characteristics and serum molecular markers and auto-antibodies of the patients

	Neu	Lymph	NLR	CRP	ESR	LDH/N
**Overall**	3650 (*278,2)*	1571 *(118,4)*	2,609 *(0,22)*	2,434 *(0,4)*	68 (5.3)	1,481 *(0,16)*
**Wt-k-ras**	3456 (507,9)	1581 (41,7)	2,512 (0,45)	1,832 (0,5)	56,5 (6.8)	1,365 (0,26)
**Mut-k-ras**	3939 (368,7)	1636 (171,7)	2,748 (0,3)	3,104 (0,8)	68 (7.8)	1,601 (0,2)

	CEA	c-ANCA	p-ANCA	ENA
**Overall**	122,5 (211.8)	0,921 *(0,1)*	0,688 *(0,08)*	0,324 *(0,04)*
**Wt-k-ras**	180.5 (383.3)	1,086 (0,1)	0,729 (0,21)	0,337 (0,02)
**Mut-K-ras**	122,5 (127.8)	0,763 (0,08)	0,625 (0,08)	0,326 (0,05)

	CD4^+^	CD8^+^	T_reg_	T_cm_	T_em_	c-NK	MDSCs
**Overall**	40,1 (*2,6)*	26,9 (*1,7)*	3,2 *(0,44)*	6,3 (*1,39)*	40,3 (*3,26)*	9.63 (*3,36)*	3.95 (*1,97)*
**Wt-k-ras**	38,2 (1,8)	25,7 (2,1)	3,2 (0,05)	4,7 (0,09)	44,7 (2,2)	7.57 (4,45)	4.47 (1,47)
**Mut-K-ras**	44,2 (3,3)	29,6 (2,8)	3,5 (0,7)	8,57 (2,67)	37,2 (5,06)	11.7^*^ (4,13)	3.55 (2,1)
						^*^0.030913	

**Table 3:** Statistical evaluation of the correlation between clinical/tumor-associated markers and the clinical outcome of the patients

Comparative marker	Cut-off value	Number of patients	Months ± SD	Endpoint	*P* value
ECOG	≤1 >2	29 10	**9,31 ± 2,27** **2,41 ± 0,43**	PFS	**<0.001**
ECOG	≤1 >2	29 10	17,04 ± 2,97 3,91 ± 0,74	OS	**<0.001**
CEA	≤ median value >median value	19 17	11,05 ± 3,36 4,41 ± 0,78	PFS	0.189
	≤ median value >median value	19 19	19,10 ± 4,19 7,42 ± 1,14	OS	**0.021**
Sex	Male Female	18 21	5.8 ± 7.2 8.05 ± 11.6	PFS	>0.25
	Male Female	18 21	11.44 ± 10.1 11.8 ± 12.2	OS	> 0.25
Age (years)	<50 ≥50	7 32	3.71 ± 2.28 7.75 ± 10.59	PFS	>0.25
	<50 ≥50	7 32	10.7 ± 5.43 11.8 ± 12.11	OS	>0.25
HLA-A2	Positive Negative	15 20	7,46 ± 2,98 7,10 ± 2,00	PFS	0.764
	Positive Negative	15 20	12,16 ± 3,35 13,70 ± 2,93	OS	0.684
K-ras status	Wild Type Mut	22 19	8,77 ± 2,29 4,68 ± 1,58	PFS	**0.050**
	Wild Type Mut	22 19	15,98 ± 3,35 8,64 ± 2,31	OS	0.160

Legend: ECOG, Eastern Cooperative Oncology Group performance status; CEA, Carcino-Embryonic Antigen; Mut, Mutated.

**Figure 2:**
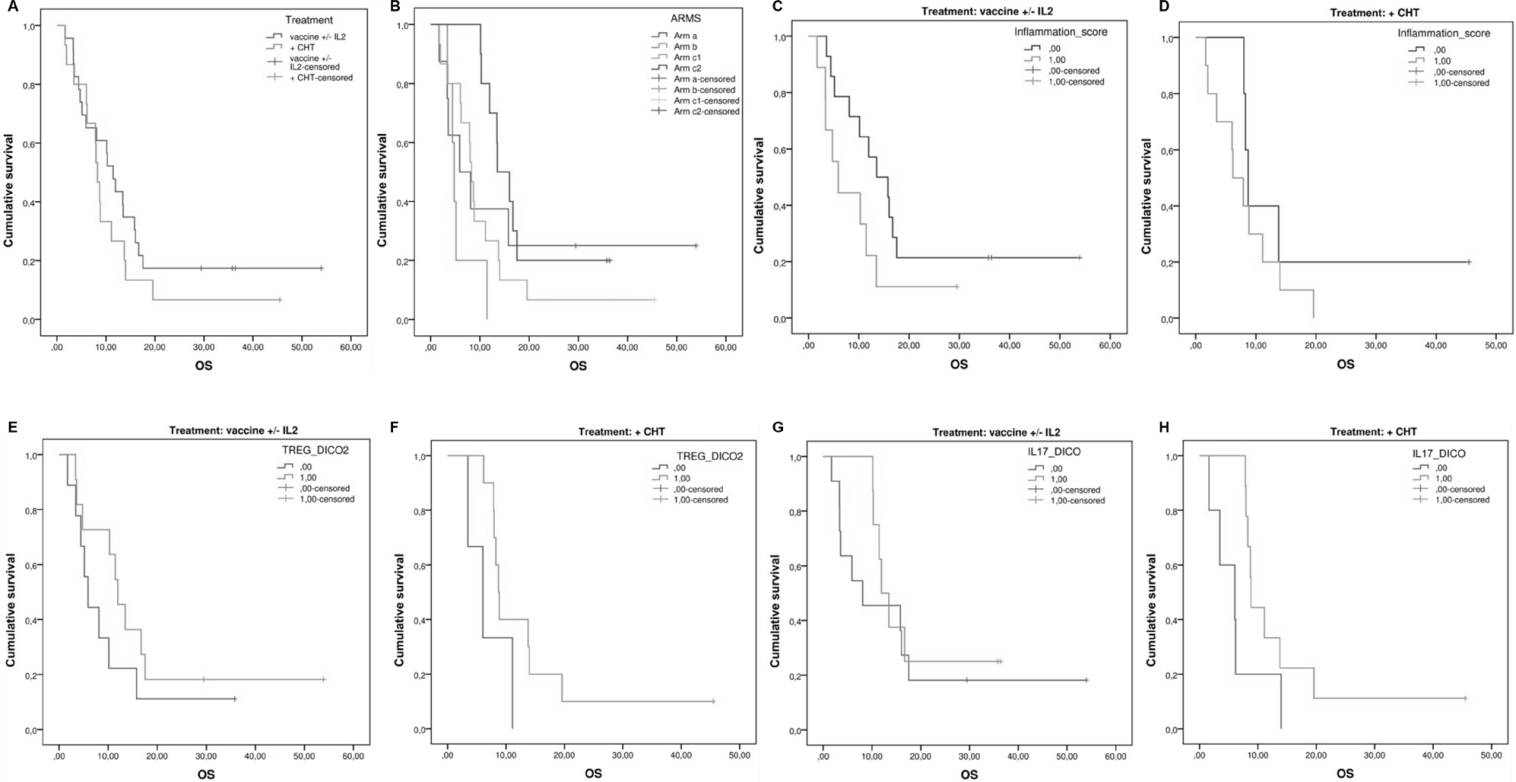
Evaluation of predictive markers in patients who received TSPP ± cytokines and TSPP + GOLFIG chemo-immunotherapy (**A**) Overall survival in mCRC patients who received TSPP ± cytokines (Arm A + B) vs those who received TSPP + GOLFIG regimen (Arm C/DL + Arm C/DL1-3). (**B**) Overall survival in mCRC patients enrolled in the different treatment arms (Arm A vs B vs C/DL0 vs C/DL1-3). (**C**) Influence of the inflammation score (NLR, PCR, LDH) on the survival of mCRC patients who received TSPP ± cytokines (Arm A and B). (**D**) Influence of the inflammation score (NLR, PCR, LDH) on the survival of mCRC patients who received TSPP + GOLFIG regimen (Arm C/DL0-3). (**E**) Influence of peripheral T_reg_s’ baseline levels on the survival of mCRC patients who received TSPP ± cytokines (Arm A and B). (**F**) Influence of peripheral T_reg_s’ baseline levels on the survival of mCRC patients who received TSPP + GOLFIG regimen (Arm C/DL0-3). (**G**) Influence of IL17/A baseline levels on the survival of mCRC patients who received TSPP ± cytokines (Arm A and B) (**H**) Influence of IL17/A baseline levels on the survival of mCRC patients who received TSPP + GOLFIG regimen (Arm C/DL0-3). Asterisk (^*^) represents statistical significance between the arms (*P <* 0.05).

Patients in the arm C/DL-0 (GOLFIG chemo-immunotherapy followed by TSPP vaccine) showed the longest survival over the other treatment groups [arm C/DL0 vs arm A, B, and C/DL 1-3; log Rank test, *p =* 0.03 and Breslow (Generalized Wilcoxon) test, *P =* 0.021] even though these results should be taken cautiously for the very small statistical sample (Figure 2B). Our statistical analysis also revealed that patients’ PFS and OS correlated with lower baseline levels of neutrophils, NLR, CRP, ESR, LDH/NV, ENA and IL4. PFS and OS also correlated with no post-treatment increases (FBV > 1) of the same inflammatory markers. Finally, a prolonged survival was also recorded in patients presenting a treatment-related increase (FBV > 1) of pANCA and highly cytotoxic NK (CD56^dim^CD16^brigth^) cells (Table 4). On the overall population, we were unable to find any significant correlation of either PFS or OS with the baseline values and post-treatment changes of TS-specific CTL precursor frequency, TNFα, IL12p70, IFNγ, IL10,IL17, CD4^+^, CD8^+^,T_cm_s,T_em_s, T_reg_s, DCs, MDSCs (Table 4 and data not shown).

**Table 4:** Correlation between immunological characteristics and inflammation serum markers levels and PFS and OS

Comparative marker	Cut-off value	Number of patients	Months ± SD	Endpoint	*P* value
NLR (neutrophil to lymphocytes ratio)	≤median value >median value	20 20	11,10 ± 3,18 3,80 ± 0,59	PFS	**0.010**
	≤median value >median value	20 20	19,01 ± 3,97 7,40 ± 1,04	OS	**0.011**
NeutrophilFBV	≤1 >1	25 14	10,16 ± 2,57 3,07 ± 0,67	PFS	**0.003**
	≤1 >1	25 14	18,51 ± 3,38 5,85 ± 1,29	OS	**0.001**
CRP	≤median value >median value	20 20	11,25 ± 3,17 3,65 ± 0,54	PFS	**0.005**
	≤median value >median value	20 20	19,75 ± 4,10 7,40 ± 1,48	OS	**0.002**
CRP FBV	≤1 >1	6 6	5,33 ± 1,05 12,83 ± 3,59	PFS	**0.019**
	≤1 >1	6 6	10,66 ± 2,00 22,04 ± 3,81	OS	**0.027**
ESR	≤median value >median value	19 18	10,15 ± 3,14 5,27 ± 1,69	PFS	0.090
	≤median value >median value	19 18	18,92 ± 3,69 7,52 ± 1,97	OS	**0.002**
LDH/LDHNR	≤median value >median value	19 19	10,84 ± 3,36 4,42 ± 0,80	PFS	0.106
	≤median value >median value	19 19	19,64 ± 3,88 6,60 ± 0,84	OS	0.001
ENA	≤median value >median value	20 17	18,47 ± 3,78 8,58 ± 2,11	OS	**0.011**
cANCA	≤median value >median value	18 18	16,66 ± 3,72 12,30± 2,99	OS	0.510
pANCA	≤median value >median value	16 18	18,37 ± 4,05 11,57 ± 2,89	OS	0.17
pANCA FBV	≤1 >1	8 13	6,12± 1,67 16,76 ± 4,22	OS	**0.039**
IFN ɣ	≤median value >median value	16 16	11,68 ± 3,07 18,31 ± 3,81	OS	0.154
IL12p70	≤median value >median value	17 17	18,05 ± 4,24 10,91 ± 1,98	OS	0.250
IL17	≤median value >median value	18 18	10,16 ± 2,57 17,83 ± 3,36	OS	**0.056**
IL10	≤median value >median value	16 16	15,19 ± 4,12 11,77 ± 1,92	OS	0.747
IL4	≤median value >median value	18 16	10.33 ± 2.80 18.99 ± 3,55	OS	**0.028**
T_EM_	≤median value >median value	19 17	13,25 ± 2,30 15,86 ± 4,46	OS	0.887
T_CM_	≤median value >median value	18 18	12,05 ± 3,42 15,64 ± 2,88	OS	0.149
T_REG_	≤median value >median value	17 19	13,94 ± 3,77 14,34 ± 2,84	OS	0.833

Legend: PFS, progression-free survival; OS, overall survival; NeutrophilFBV, Neutrophil fractional blood volume; CRP, C-reactive protein; ESR, erythrocyte-sedimentation rate; LDH/LDHNR, Lactate Dehydrogenase/Lactate Dehydrogenase Normal Range; ENA, anti-extractable nuclear antigen; cANCA, anti-neutrophil cytoplasmic antibodies/ anti-myeloperoxidase; pANCA, anti-neutrophil cytoplasmic antibodies/anti-proteinase-3; IFN ɣ, Interferon gamma; IL, interleukin, T_EM_, effector/memory T lymphocytes; T_CM_, central memory T lymphocytes; TREG, regulatory T lymphocytes.

### Predictive markers in patients who received TSPP ± cytokines and TSPP + GOLFIG chemo-immunotherapy

Then, the predictive values of these inflammatory and immunological parameters was separately investigated in patients receiving TSPP vaccine ( ± cytokines) alone or together with cytotoxic drugs. The two groups did not show difference in term of survival [arm A + B vs C/DL0 + C/DL1-3: 13.49 ± 4.89 (95% CI 3.92–23.07) *vs.* 14.62 ± 2.45 (95% CI 9.82–19.42); *p* = 0.29] (Figure 2A). On the other hand, those patients who received the sequential treatment (arm C/DL0) with GOLFIG followed by TSPP vaccination showed the longest survival over the other groups (*p* = 0.03) (Figure 2B). First of all, a longer survival in patients with lower NLR, independently by the treatment arm, was confirmed (*p* = 0.016) (Figure 2C–2D). We also reported that a lower T_reg_ expression at baseline showed a trend to longer survival in both groups (arm A + arm B: *p* = 0.062) (arm C DL1-3 and DL0: *p* = 0.088) (Figure 2E–2F). Moreover, we observed that higher IL17/A levels at baseline correlated with longer survival in the group of patients enrolled in the arm C (DL0-3: *p =* 0.017) only, with no correlation in the other group (arm A + B: *p* = 0.253) (Figure 2G–2H). In order to identify specific inflammatory immunological signatures predictive of response to TSPP vaccination we assembled and tested an inflammation-score. It was composed by assigning an arbitrary value to specific inflammatory parameters at baseline. Each parameter (ESR, CPR and LDH/NV) was categorized as zero, when it was < the specific median value and 1 if it was ≥ the median value. The inflammatory score derived from the sum of the three parameters’ values. When a statistical analysis was carried out a low inflammation-score (<2) was highly predictive of longer survival in patients enrolled in arm A and B [high *vs.* low score (<2) high vs. low score: *p =* 0.049) while it showed no significance in patients enrolled in the arm C (high vs. Low score: *p* = 0.199) (Figure 2B).

### Predictive markers in mCRC patients with k-ras/wt and k-ras/mut who received TSPP vaccine

Finally our analysis revealed that either inflammatory or immunological parameters, and independently by the treatment arm, showed substantial differences when respectively, correlated with OS in patients bearing k-ras/mut.

In particular, in the latter group of patients the predictive value of ESR, CRP, LDH/NV and ENA levels at baseline was lost in term of survival.

**Figure 3:**
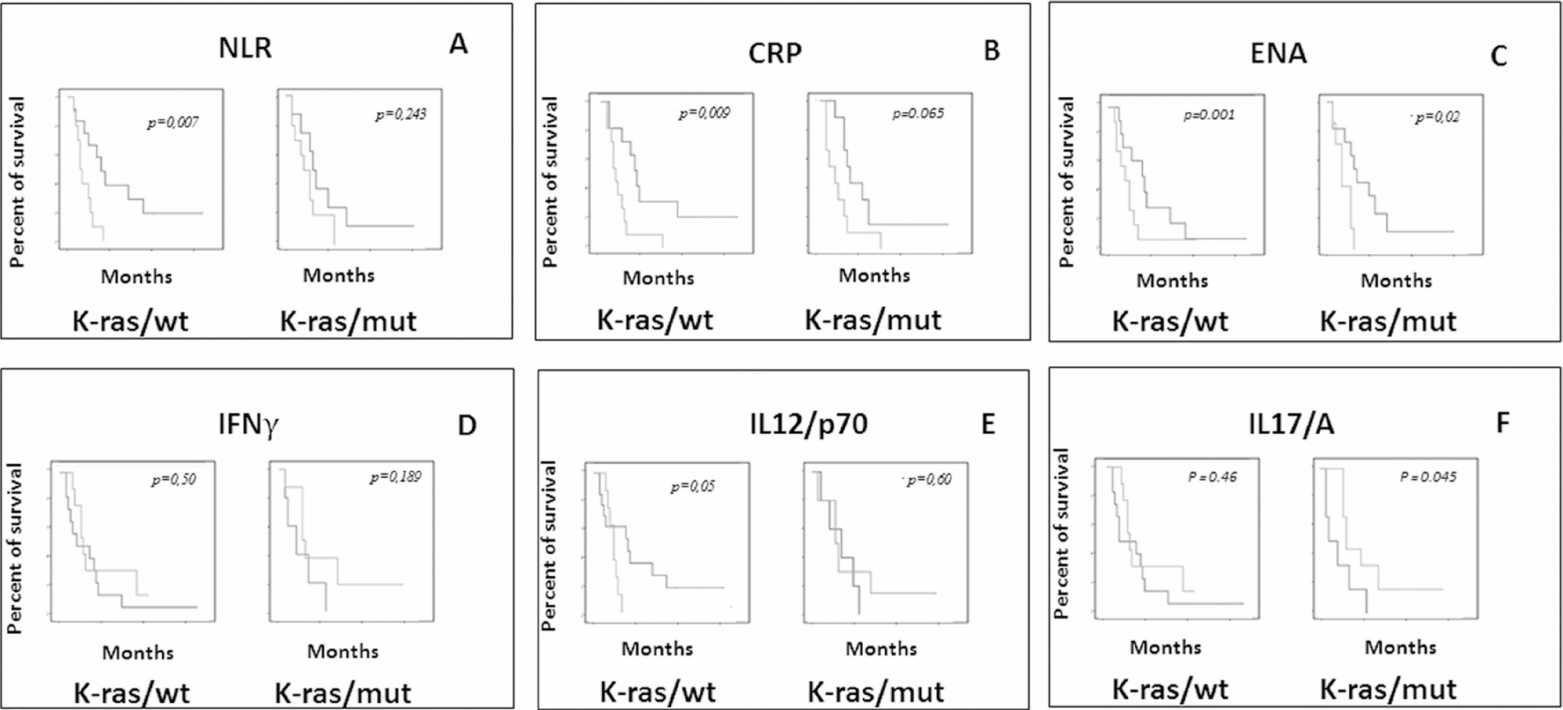
Evaluation of predictive markers in mCRC patients with k-ras/wt and k-ras/mut who received TSPP vaccine Different influence of baseline levels of NLR (**A**), CRP (**B**), ENA (**C**), IFNγ (**D**), IL12/p70 (**E**) and IL17/A (**F**) on k-ras wt and k-ras mut patients treated with TSPP vaccine. Overall survival was compared between the two groups of patients with baseline levels < and ≥ the median value of each specific parameter. Asterisk (^*^) represents statistical significance between the arms (*P <* 0.05).

Additionally, we found the OS of patients with k-ras/wt, and not those with k-ras/mut, was specifically correlated with baseline IL12/p70 levels lower than the median value (*p =* 0.034), while the OS of patients with k-ras/mut, and not those with k-ras/wt, specifically correlated with the baseline IL17/A levels over the median value (*p =* 0.01) (Table 5 and Figure 3).

**Table 5:** Inflammatory predictive markers/k-ras mutational status correlation with clinical outcome of the patients

Subgroup	Comparative marker	Cut-off value	Number of patients	Months ± SD	Endpoint	*P* value
**k-ras/wt**	Neutrophil Count	≤median value >median value	10 9	24,11 ± 6,21 10,22 ± 2,72	OS	**0.050**
	NLR	≤median value >median value	12 8	23,06 ± 5,37 8,25 ± 1,57	OS	**0.020**
	CRP	≤median value >median value	11 9	23,26 ± 5,62 9,44 ± 2,65	OS	**0.023**
	ENA	≤median value >median value	9 10	24,46 ± 5,55 10,90 ± 3,36	OS	**0.029**
	IL12 p70	≤median value >median value	12 7	22,58 ± 5,45 9,71 ± 1,10	OS	**0.034**
	IL17	≤median value >median value	11 8	14,54 ± 4,03 21,50 ± 5,35	OS	0.379
**k-ras/mut**	Neutrophil Count	≤median value >median value	9 9	7,77 ± 2,29 10,00 ± 3,04	OS	0.567
	NLR	≤median value >median value	8 10	10,75 ± 3,48 7,10 ± 1,70	OS	0.411
	CRP	≤median value >median value	9 9	12,55 ± 3,45 5,77 ± 1,88	OS	0.123
	ENA	≤median value >median value	11 7	11,48 ± 3,06 5,28 ± 0,91	OS	0.055
	IL12 p70	≤median value >median value	5 10	7,60 ± 3,01 11,75 ± 3,26	OS	0.247
	IL17	≤median value >median value	6 9	4,50 ± 2,46 14,27 ± 3,10	OS	**0.010**

Legend: OS, overall survival; NLR, Neutrophil/lymphocyte ratio; CRP, C-reactive protein; ENA, anti-extractable nuclear antigen; IFN ɣ, Interferon gamma; IL, interleukin.

## DISCUSSION

The results of the TSPP/VAC 1 trial suggested that TSPP vaccination is safe, induces immune-modulatory effects and exerts antitumor activity in 41pretreated mCRC patients (third/fourth treatment line), who showed a PFS and OS of 6.9 and 14.9 months, respectively. These results can be considered very encouraging at the light of the fact that Regorafenib, the multi-kinase inhibitor currently recommended for salvage treatment of pretreated mCRC, is associated to a PFS and OS, respectively of 2 and 6.4 months, with significant adverse events and very high costs [12]. These results in mCRC patients may also be of interest considering that mCRC-immunotherapy has been a dynamic field of investigation for more than 30 years with controversial results in term of antitumor activity [13, 14]. In fact, results of several immunotherapy trials have shown that it is possible to use different antigen vectors to trigger an efficient T cells response to tumor associate antigens such as CEA, MUC-1, k-ras/mut in mCRC patients [13]. Nevertheless, even in the presence of a significant treatment-induced T cell response, none of these agents has been able to improve the outcome of these patients [13]. Several reasons have been advocated to explain these negative results, including the poor immunogenicity of the target antigens and/or failures in the delivery constructs, occurrence of immune-suppressive cell lineages (T_reg_s, MDSCs, M2-macrophages, etc.), the resistance of CCR cells to the immune-effectors, the interference of multiple immune-checkpoint inhibitors, and finally, a tumor-protective-microenvironment associated with CRC [13]. The latter points in particular have been associated to chronic inflammation, neo-angiogenesis and hypoxia, infiltration by T_reg_s, MDSCs, and other immune-suppressive cell lineages expressing PDL-1 and PDL-2 [15]. More recent immunotherapy approaches involving immune-checkpoint blockade, successful in the treatment of other solid tumors, have instead achieved deluding therapeutic results in mCRC patients [16–20]. In fact, mAbs to CTLA-4 resulted completely inactive [15–16], while mAbs to PD-1/PDL1 immune-checkpoint have shown antitumor activity only in a subset of patients with deficient mismatch repair (dMMR). In the latter case, it has been hypothesized that the dMMR status increases the burden of neo-antigens that elicit a very proficient CTL response with potential antitumor activity susceptible to PD-1/PDL1 axis, and consequently, to mAb blockade [16]. All together, these results clearly suggest that immune-checkpoint blockade requires the presence of highly cytotoxic CTLs educated to destroy mCRC cells expressing critical antigens, eventually inhibited in the tumor tissue throughout PD-1 axis, as main mechanism of immune-escape. In this context, the use of TSPP vaccination to elicit a TS specific CTL response prior scheduling PD1/PDL-1blockade could be a very successful treatment strategy to take in consideration for mCRC patients. Additionally, the results of several studies suggest that cytotoxic drugs like oxaliplatin, gemcitabine, 5-FU or cyclophosphamide, mAbs to EGFR and VEGF, as well as radiotherapy, may induce immunogenic cell death, and shape mCRC micro-environmental conditions making the residual tumor tissue more sensitive to activated immune-effectors [17–20]. On the other hand, previous clinical results of our group on the GOLFIG regimen have shown that a rationale combination of chemotherapy and immune-adjuvant cytokinesis are really capable to improve both PFS and OS in mCRC [8–11]. The GOLFIG regimen has been designed to mimic an *in vitro* procedure to generate very efficient CCR-specific CTLs from human PBMCs and in parallel to shape the residual tumor to become more susceptible to the immune-effector activity [8, 13, 21]. In preclinical models, this multi-drug regimen was able to induce immunogenic cell death in CRC cells, with massive release of TAAs in a context of danger signal and cross-priming, which in turn was able to trigger a multi-antigen specific T cell response with potent antitumor activity [8, 21]. In line with these preclinical results, two consecutive phase II and phase III trials showed that the use of this regimen in mCRC patients, elicits a CEA/TS specific T cell response, increases the amount of peripheral and central T_cm_s, and in parallel decreases the score of tumor infiltrating T_reg_s [9–11]. Sixteen percent of the mCRC patients receiving this treatment, presented autoimmunity, an event which resulted strongly predictive of prolonged PFS and survival [10]. Based on these immune-adjuvant and immune-shaping properties, the GOLFIG regimen was investigated in concomitant and sequential combination with TSPP in the arm C of TSPP/VAC-1 trial in mCRC patients (arm C/DL1-3 and C/DL0, respectively). Even though the three arms were not designed on comparative setting, those patients who had received TSPP vaccination after multiple GOLFIG courses (arm C/DL0) showed a very promising outcome, with a PFS and OS of 7 and 16 months, respectively [5, 6]. In line with these data, the results of our study in mCRC patients undergone TSPP vaccination suggested that the number of previous treatments, age, gender, treatment arm (A, B or C), HLA-A(*)02.01 haplotype, TS levels and TIL immune-phenotype in the tumor, at baseline, were not able to influence the outcome of these patients. On the other hand, a good performance status and lower CEA levels at baseline, representative of a smaller tumor burden, were associated to a better outcome. Additionally, the presence of an activating K-ras mutation was associated to a worse outcome in term of PFS and to a trend to a worse survival. Concerning the immune-biological monitoring, the best outcome in patients with lower systemic inflammatory baseline profile (neutrophil counts, NLR, CRP,ESR, LDH/LDHNV, and ENA) and higher baseline levels of IL-4, which may promote occurrence of auto-antibody-driven autoimmunity, was found [22, 23]. The best outcome was also detected in vaccines with increase in pANCA, an auto-antibodies, whose presence is suggestive of autoimmunity and micro-vascular damage [24]. A prolonged survival was found in patients who showed a progressive rise in peripheral CD3^-^CD56^dim^CD16^bright^NKs, a lymphocyte subset, which together with an antigen independent antitumor activity also holds the ability to promote an antigen specific T cell response stimulating the functional activity of peripheral DCs [25]. In our study, other parameters, including cytokines’ (TNFα, IL17/A, IL12/p70, IL10) and cell subsets’ (CD3^+^CD4^+^, CD3^+^CD8^+^, T_cm_s, T_em_s, T_reg_s, MDSCs) in peripheral blood, did not correlated with either PFS or OS on the whole population. Since experimental evidence suggests that alterations on k-ras pathway in CRC cells may have dramatic consequences within tumor micro-environment, involving inflammation, angiogenesis, and immune-response, we examined the predictive values of our markers in the two subsets of patients with k-ras/wt and k-ras/mut. The results showed that the inflammatory markers have a strict correlation with survival only in the k-ras/wt group, losing any statistical significance in patients with k-ras/mut. Interestingly, in the latter group of patients, the outcome was directly correlated to the baseline levels of IL17/A, an inflammatory cytokine able to amplify and empower the cytotoxic effects of pre-existing CTLs in CRC tumor sites [26]. These 2 groups of patients did not show significant difference prior TSPP treatment with the exception of a much lower tumor infiltration by CD15^+^ inflammatory cells in the k-ras/mut group. This finding is suggestive of a different cancer associate-inflammatory profile within the tumor tissue with a different sensitivity to both cytotoxic stimula and cytotoxic effectors. In line with these findings, the 2 groups of patients presented a different immune-biological response to TSPP vaccination. In fact, patients with k-ras/mut failed to induce auto-antibodies, such as cANCA and ENA, in response to TSPP vaccination, while their treatment was associated to increase in peripheral levels of IFNγ, IL17/A, and T_reg_s. In this context, IFNγ is an inflammatory cytokine, which is able to enhance the expression of PDL-1 on CRC cells and immune-cells in the tumor sites. This event potentially leads to empowerment of the PD-1/PDL1 immune-check point activity and neutralization of activated CTLs in the tumor. Additionally, and it is also known that IL17/A together with IFNγ promotes the switch of inactive T_reg_s to highly suppressive T_reg_s [27], which in turn, could affect the primary T cell response to TSPP vaccine of these patients. On the other hand, the results of other studies suggest that IL17/A production may also empower the cytotoxic activity of pre-existing effector CTLs in the tumor site [28].This finding could also partially explain the predictive value of IL17/A baseline levels only in patients with k-ras/mut and in patients who receive TSPP vaccination and GOLFIG chemo-immunotherapy (Arm-C/DL0-3).

A number of studies have already, highlighted the detrimental effects of systemic inflammation in patients with different malignancies undergoing immune-biological treatments [29–35]. Several studies, also suggest the existence of a strong link among angiogenesis, pro-inflammatory context and immune-editing within the tumor environment, which in turn could affect the responsiveness of the malignant cells to pro-apoptotic signals and immune-response [36].

These detrimental effects of cancer-associate inflammation are similar to those induced in the tumor microenvironment by the presence of a malfunctioning k-ras pathway. In this context, it has been shown that the expression of activating k-ras mutation in CRC cells promotes the release pro-angiogenic factors, pro-inflammatory cytokines, and chemokines, that in turn, make the tumor micro-environment able to protect the tumor by multiple pro-apoptotic stimula (cytotoxic drugs, anti-EGFR mAbs, CTLs, etc.) [37–43]. This hypothesis is in line with the finding that patients with k-ras/wt and k-ras/mut present a different immune-biological response to TSPP vaccination with a worse outcome.

On these bases, we can conclude that the outcome of mCRC patients vaccinated with TSPP may be greatly hampered by their systemic inflammatory profile and by an altered k-ras pathway. This finding deserves further studies and must be taken into account in the design of future studies aimed to evaluate the antitumor activity of immunological strategies like TSPP vaccine in mCRC patients.

## PATIENTS AND METHODS

The TSPP/VAC-1 is a phase Ib trial program designed to test in advanced cancer patients, the toxicity and immunological activity of TSPP in different therapeutic conditions,. The protocol consisted of three parallel and independent arms where TSPP vaccination was administered alone (arm-A) and in combination with the immune-adjuvant IG-1 regimen (arm-B) and in combination with the chemo-immunotherapy GOLFIG regimen (arm-C). The latter arm was reserved to mCRC patients and evaluated the effects of peptide vaccination, administered concomitantly (DL1-3) or after (DL0) 10/12 GOLFIG courses.

### Ethical considerations and study design

The study was designed according to good clinical practice (GCP) recommendations. It was authorized by the Italian National Institute of Health (Istituto Superiore di Sanità), the Italian Ministry of Health, and approved by the University of Siena Ethical Committee Board (equivalent to Human Subject Committee of Investigational Review Board). The study registered with the TSPP/VAC-1 code (Eudract: # 2009-016897-33)was planned as a dose escalation trial, in three parallel and independent arms (A, B and C). TSPP dose-escalation was planned according to the Fibonacci’s series. The first cohort of patients received 100 µg of peptide (DL1), the second, 200 µg (DL2), and the third 300 µg (DL3), every 21 days. New patients could be enrolled in higher dose level cohorts, only if no Grade IV event was demonstrated in patients treated with lower doses. Patients of arm A (8) received vaccine peptide alone, those of arm B (4) received TSPP and sc. GM-CSF (Sargramostim / Leukine^®^, Berlex, USA) (50 µg days 1–5) and sc. Aldesleukine/Proleukin^®^, Novartis, Switzerland (0.5 MIU bi-daily, days 6–15) according to the previously described IG-1 schedule [7]. Patients of arm C/DL1-3 (17) received peptide vaccination seven days after the beginning of the chemo-immunotherapy cycle with gemcitabine 1g/sqm on the day 1, oxaliplatin 85 mg/sqm on the day 2, Levofolinic acid 100 mg/sqm on the days 1 and 2, bolus 5′-FU 400 mg/sqm on days 1 and 2 and infusional 5′-FU 800 mg/sqm on days 1 and 2, every 15 days according to the previously described GOLFIG [9–11]. These patients also received sc. GM-CSF (50 µg days 3–7) and sc. IL-2 (0.5 MIU bi-daily on days 8–14 and 17–29). In particular, 17 patients of the latter group, received sc. TSPP vaccination at escalating dosage [3 patients entered the DL-1; 3, the DL-2; 11, the DL-3 cohort] on biweekly bases, starting one week after each chemotherapy cycle (concomitant treatment). Other12 patients received GOLFIG chemo-immunotherapy alone (DL0) for 10/12 courses and then maintenance therapy with the same schedule adopted for arm B (TSPP + IG1).

Two patients of the DL0 group did not receive TSPP vaccination, due to early disease progression and decline in performance status, thus they were excluded by the statistical analysis. The remaining 10 patients entering the maintenance therapy group (DL0_mant_), received TSPP vaccination every 3 weeks (300 µg on the day 1), sc.GM-CSF (50 µg at day, days 1–5 every 3 weeks), and sc.rIL2 (0.5 MIU twice at day, days 6–15 every 3 weeks) [5, 6].

### TSPP Vaccine

TSPP (YMIAHITGLFLDSLGFSTTLGDAHIYL) [4] was synthesized and characterized by good manufacturing practice (GMP) procedures by the American Peptide Ltd (Rockville, MD,USA). The aseptic vial filling process was performed by the Pharmacy of the Azienda Ospedaliera Universitaria Senese, which also performed the stability study, endotoxin evaluation and chemical related toxicity analysis of the product. TSPP was dissolved in DMSO and the exact peptide dose (100, 200 or 300 µg) was diluted with PBS in a volume of 250 µl, and then 1:2 diluted with Montanide ISA 720 VG ST (Seppic, Milan, Italy) as adjuvant. The final volume of the vaccine was 500 µl/dose.

### Patients’ population and study endpoints

Our analysis was performed on a sample of 41 mCRC patients. The primary endpoints of the study were the identification of the maximal tolerated dose (MTD) and the most effective biological dose (MEBD) of TSPP peptide, by evaluating the frequency of adverse events per dose level and predefined immune-biological events in the three cohorts of patients. Evidence of anti-tumor activity was a secondary endpoint. The inclusion criteria were: written informed consent concerning treatment risk and biological monitoring, histological diagnosis of malignant disease, at least two previous chemotherapy lines for advanced disease, measurable disease (according to WHO tumor response criteria), ECOG performance status ≤1, normal renal and hepatic functions, white blood cell count ≥2,500/mm^3^, hemoglobin levels ≥9 g/dl, platelet cell count ≥100,000/mm^3^, and normal heart function. The exclusion criteria were: any major organ failure, central nervous system involvement, second malignancies, active infectious disease, major inflammatory and autoimmune rheumatic diseases, and acquired immune-suppression. Treatment allocation was not masked. Standard clinical and laboratory evaluation (clinical history, physical examination, blood count and chemistry, serum dosage of C-reactive protein (CRP), erythrocyte-sedimentation rate (ESR), lactate dehydrogenase (LDH), rheumatoid factor, carcinoembryonic antigen (CEA) and CA19.9 assays, chest x-rays and ultrasound abdominal scans, were performed at baseline and repeated every six weeks. Patients’ sera were tested for antinuclear antibodies (ANA) by IFA, (starting dilution 1:160) (SSA HEp2000, ImmunoConcepts); EliASymphony screening (Thermo Fisher Scientific); further ELiA tests were performed for single ANA specificities. ANCA, p-ANCA, and C-ANCA were measured by indirect immunofluorescence using INOVA substrate, while ENA was tested on Phadia250 instrument. Contrast CT scans were scheduled every three months. Patients enrolled in the arm C/DL1-3 received combined TSPP/GOLFIG treatment for a maximal of 12 cycles, then those who did not progress, received TSPP vaccination every 21 days at the dosage of 300 µg until disease progression, occurrence of unacceptable toxicity, clinical judgment, or withdrawal of consent. Any further treatment decision after disease progression was left to the physician in charge. Adverse events, toxicity and treatment response were evaluated according to WHO classification.

### ELISA assays and multiplex analysis

Serum levels of Interferon(IFN)-ɣ, Tumor necrosis factor (TNF)-α, IL10, IL4, IL12/A, IL10 and IL17/A cytokines were measured using Bio-Plex human cytokine multiplex kits (Bio-Rad Inc., Hercules, CA). Briefly, a standard curve was created via dilution of premixed standards to 50,000 pg/ml, followed by serial dilution to 8 concentrations ranging from 32,000 to 1.95 pg/ml. The assay was performed in the 96-well filtration plate supplied with the Bio-Plex kit. Premixed beads coated with target antibodies (50 µl) were added to each well, and then washed twice with Bio-Plex wash buffer. Premixed standards or undiluted samples (50 µl) were then added to the wells, followed by shaking at 1,100 rpm for 30 sec and incubation for 30 min with shaking at 300 rpm at room temperature. Wells were then washed 3 times with Bio-Plex wash buffer, and 25 µl of the premixed detection antibodies was added to the wells. This was followed by shaking at 1,100 rpm for 30 sec and incubation for 30 min with shaking at 300 rpm at room temperature. Wells were again washed 3 times with Bio-Plex wash buffer, and 50 µl of streptavidin-PE was added to the wells. This was incubated for 10 min with shaking at 300 rpm. Wells were washed 3 times with Bio-Plex wash buffer, and the beads were resuspended in 125 µl Bio-Plex assay buffer. The samples were then read using the Bio-Plex suspension array system. The fluorescence intensity of the beads was measured by using the Bio-Plex array reader. Bio-Plex Manager software with five-parametric-curve fitting was used for data analysis.

### Fluorescence-activated cell sorting analysis of patient peripheral blood mononuclear cells (PBMC)

The patients’ PBMCs were purified by Ficoll-Hypaque (Celbio S.P.A., Italy) gradient separation of buffy coats of heparinized blood samples and analyzed by Fluorescence-activated cell sorting (FACS) analysis, as described in previous studies [5, 6].

PBMC were stained with different pools of labeled monoclonal antibodies (mAbs) (CD4V450, CD45RAPE, CD62LFITC, CCR7PE-Cy7, Pharmingen;CD8PerCPCy 5.5, CD45ROAPC, CD3FITC, CD19FITC, CD14FITC, CD11cAPC, CD16PE, Rat IgG2aFITC, Mouse IgG1, Becton Dickinson, Italy; CD56 APC, Mouse IgG2a APC Immunotools, DE; CD15 PE ABCam, UK; CD25 PE, FoxP3 FITC, eBioscence, UK) and examined by a FACScalibur BD instrument. The fluorescent-minus-one and isotype control were included in each experiment in order to appropriately set the gates. Ki67 positive cells and T_reg_s were analyzed after intracellular immune-staining according to the manufacturer’s protocol (eBioscience). Cytofluorimetric analysis was carried out by using the FlowJo^®^ software. The mean fluorescence intensity (MFI) and percent of positive expression (%) of each marker were measured. Results were expressed as fold induction relative to baseline indicated as 1.

### Immunoistochemistry

immunohistochemistry was performed for TS expression in primary tumors and tumor infiltrating T cells expressing FoxP3 (Treg), CCR7 (Tcm/em), CD4, or CD8 and inflammatory cells expressing CD15. This analysis was carried out in the primary tumor of 41 mCRC patients who received TSPP vaccine, whose 22 with k-ras wt and 19 with mutated k-ras. In brief, sections were deparaffinized and peroxidase activity blocked. Antigen retrieval was performed with 1 mμ EDTA pH 8.0, following which endogenous biotin was blocked by use of a commercial kit (Vector Laboratories, Inc., Burlingame, CA, USA) before incubation in 20% swine serum for 30 min. The primary antibody (#9718; Cell Signalling Technology, Inc., Danvers, MA, USA) was added at a concentration of 1 : 100 and sections incubated overnight at 4° C. Sections were then incubated for 90 min with a biotin-conjugated secondary antibody (Dako UK Ltd, Ely, UK) and for 45 min with streptavidin biotin-peroxidase conjugate (Vector Laboratories, Inc.). 3,3′-diaminobenzidine tetrahydrochloride (DAB; Vector Laboratories, Inc.) was used as the chromogen. The numbers of positive stained cells in 15 separate high-power fields (HPF; magnification ×20) were counted in a blinded manner. Results are expressed as number of positive cells per HPF.

### Statistical analysis

Survival analysis and correlations between patient’s baseline characteristics and toxicity were evaluated by Kaplan Meier curves and Wilcoxon test statistic in the univariate analysis. All potential prognostic factors were transformed into categorical variables. Results were expressed as mean ± standard deviation (SD) of three determinations made in three different experiments, and analysed by the 2-tail Student’s *t*-test. A *p*-values ≤ 0.05 were considered statistically significant. Variables with a *p*-value lower than 0.10 were used to construct the predictive models. These variables were entered into a multivariate analysis model according to Cox proportional hazard model to analyse the role of confounding factors, modelling the relationship among a set of one or more covariates and the hazard rate. The most significant variables were entered in the model through a step-wise method. Time variable contains the length of time during which a subject has been observed, representing a failure/censor time. Overall survival (OS) was measured from the beginning of the treatment to the day of death from any cause. Progression-free survival (PFS) was calculated from the the beginning of the treatment to the day of local or distant recurrence or death from any cause. PFS and OS in particular, were correlated with selected immune-biological parameters with potential prognostic values. In order to perform the screening of these markers we evaluated the quantitative baseline values by dividing the patients in two groups according to the specific median value of each marker prior and after treatment and median post-treatment fold change to baseline values. Univariate analyses were conducted with the log-rank test and multivariate analyses with the Cox proportional hazard model. All survival data were analysed by using the SPSS software (version 23) and GraphPadInstat 3.2. statistical packages.

## Abbreviations

ENA: anti-extractable nuclear antigen
cANCA: anti-myeloperoxidase
pANCA: anti-neutrophil cytoplasmic antibodies/anti-proteinase-3
ANA: Anti-nuclear anti-bodies
CEA: carcinoembryonic antigen
CRP: C-reactive protein
DC: dendritic cells
ECOG: eastern cooperative oncology group performance status
ESR: erythrocyte-sedimentation rate
FACS: fluorescence-activated cell sorting
GM-CSF: granulocyte macrophage-colony-stimulating-factor
LDH: lactate dehydrogenase
IFN-ɣ: Interferon
IL-2: interleukin-2
mCRC: metastatic colorectal cancer
PBMCs: peripheral blood mononuclear cells
TNF: Tumor necrosis factor
TSPP: Thymidylate-synthase poly-epitope peptide
